# Targeting IgG in Arthritis: Disease Pathways and Therapeutic Avenues

**DOI:** 10.3390/ijms19030677

**Published:** 2018-02-28

**Authors:** Kutty Selva Nandakumar

**Affiliations:** 1 School of Pharmaceutical Sciences, Southern Medical University, Guangzhou 510000, China; nandakumar@smu.edu.cn; 2 Department of Medical Biochemistry and Biophysics, Karolinska Institute, 17177 Stockholm, Sweden

**Keywords:** rheumatoid arthritis, antibodies, collagen, glycosylation, disease pathways, therapy, experimental arthritis

## Abstract

Rheumatoid arthritis (RA) is a polygenic and multifactorial syndrome. Many complex immunological and genetic interactions are involved in the final outcome of the clinical disease. Autoantibodies (rheumatoid factors, anti-citrullinated peptide/protein antibodies) are present in RA patients’ sera for a long time before the onset of clinical disease. Prior to arthritis onset, in the autoantibody response, epitope spreading, avidity maturation, and changes towards a pro-inflammatory Fc glycosylation phenotype occurs. Genetic association of epitope specific autoantibody responses and the induction of inflammation dependent and independent changes in the cartilage by pathogenic autoantibodies emphasize the crucial contribution of antibody-initiated inflammation in RA development. Targeting IgG by glyco-engineering, bacterial enzymes to specifically cleave IgG/alter N-linked Fc-glycans at Asn 297 or blocking the downstream effector pathways offers new avenues to develop novel therapeutics for arthritis treatment.

## 1. Introduction

Rheumatoid arthritis (RA) in the articular joints involves a multicellular inflammatory process; infiltration of lymphocytes and granulocytes into the articular cartilage, proliferation of synovial cells, leukocyte extravasation, and, neo-vascularization of the synovial lining surrounding the joints [1]. This proliferative process not only induces swelling, erythema, and pain of multiple joints, but also progresses to the destruction and loss of cartilage and bone architecture. Many cellular components (macrophages, dendritic cells, synovial cells, mast cells, neutrophils, T cells, and B cells), cell surface molecules (co-receptors, adhesion molecules, and integrins), signaling components (ZAP70, PTPN22, JAK, MAPK and Stat1), metabolic components, and humoral mediators (antibodies, cytokines, chemokines, metalloproteinases, serine proteases, and aggrecanases) interact and aid in the disease progression, leading to the digestion of extracellular matrix and the destruction of articular structures [2].

Several theories on the pathogenesis of RA have been put forward that are based on autoantibodies and immune complexes, T cell mediated antigen specific immune responses, cytokine deregulations, and aggressive tumor-like behavior of the rheumatoid synovia. Improved understanding of the cellular and molecular events occurring in the rheumatoid joints during the pathogenesis of the disease is particularly important to find new or better combination of therapeutics for RA [3].

The major genetic factor that is consistently associated with RA is human leukocyte antigens (*HLA*), located on chromosome 6 in the major histocompatibility complex (*MHC*) class II region, which participate in the antigen presentation. *DR* genes, including *DR4* and *DR1* are associated with RA. The susceptibility epitope is glutamine-leucine-arginine-alanine-alanine (QKRAA) or glutamine-arginine-arginine-alanine-alanine (QRRAA), the so-called shared epitope identified in amino acids 70 through 74 in the third hypervariable region of the DRβ chain [2]. In addition, Raychaudhuri et al. have identified the amino acids (leucine or valine variants at amino acid position 11) that are located in the base of the antigen binding groove as further possible explanation for antigen selection [4]. The predominance of *HLA* and prominent infiltration of T cells to the rheumatoid synovia have suggested a key role for T cells in RA. Specific peptides that bind to these DR proteins in RA patients may promote arthritis, however, so far no such dominant peptides have been identified. It is possible that the susceptibility epitope is closely linked to other genes in the MHC region, and, T cells might drive the inflammation by their cellular interactions and cytokine production [5].

On the other hand, B cells contribute to the disease pathogenesis as antigen presenting cells, through co-stimulatory functions by supporting neo-lymphogenesis as well through the secretion of antibodies [6]. In RA, autoantibodies (rheumatoid factors (RFs), anti-citrullinated protein/peptide antibodies (ACPAs)) provide diagnostic and prognostic criteria, and serve as surrogate markers for disease activity), and may play a requisite role in the disease pathogenesis (anti-CII and anti-GPI antibodies) as well. RFs have been consistently associated with RA (60–80% sero-positivity), but it has also been reported to be present in normal individuals as well as under other chronic inflammatory conditions [7]. The contributions of antibodies to the disease are not solely dependent upon their direct binding to their respective antigens, but also through indirect mechanisms, including immune complex formation, deposition, and activation of complement components and FcγRs. Modulation of circulating ICs and pathogenic antibodies by removal using therapeutic plasmapheresis [8] or depleting B cells with the antibody rituximab proved to be beneficial for RA patients [9].

Most likely candidate autoantigens in RA are the joint derived macromolecules. Arthritis can be induced in animals by immunization with different components of cartilage; collagen type II (CII), collagen type IX (CIX), and collagen type XI (CXI), proteoglycan (PG), cartilage link protein (CLP), and chitinase 3-like protein 2 (CHI3L2/YKL-39). CII, a homo-trimer composed of α1(II) chains, is the most abundant fibrillar protein that is found in the articular cartilage and constitutes 80–85% of the total collagen. Autoimmunity to CII occurs in RA, target of inflammatory attack and CII has been proposed to be the driving force in arthritis [10]. 

Immunization of susceptible rodents with CII emulsified in adjuvant induced polyarthritis (so called, collagen induced arthritis, CIA), which resembles RA in several aspects. It has been well documented that both T and B cells are important in the disease pathogenesis, as demonstrated by the resistance of mice for arthritis induction that are deficient in these cell populations [11,12]. Similar to RA, susceptibility to CIA in rodents is closely associated with the expression of specific class II molecules of the *MHC* that are involved in the specific recognition of T cell receptor (TCR) and in binding and presenting antigenic peptides to it. Mice having H2^q^ and H2^r^ haplotypes are the most susceptible to arthritis [13]. Various humanized *HLA* transgenic mice having *HLA-DQ8* [14], *DR1* [15], or *DR4* and CD4 [16] developed severe arthritis after CII immunization. In the H-2^q^ context, the dominant heterologous T cell epitope resides in the amino acids position 260–270 [17,18]. Substitution of amino acids at positions 260-264 and 266 appeared to be critical for T cell recognition [19,20]. Interestingly, epitope glycosylation is important for T cell recognition of CII in CIA [21,22].

On the other hand, major B cell epitopes well defined so far (C1, J1, U1, D3, F4, and E8) are spread over the entire triple helical CII molecule. CII reactive B cells were shown to be neither negatively selected, somatically mutated, nor tolerized [23,24]. Native but not the denatured CII induces arthritis suggests the requirement of triple helical confirmation of CII for disease induction [25,26]. In CIA, antibodies play a major role in the immuno-pathology of autoimmune arthritis, and IgG and C3 depositions were detected in the inflamed joints [27,28]. Antibodies against C1, J1, and U1 epitopes were detected in CII immunized chronic arthritis mice [29], and these CII epitopes are conserved across the species [30]. However, DBA/1 mice deficient in the *RAG1* gene still developed some synovial hyperplasia, pannus, and erosions of cartilage and bone [31], demonstrating that arthritis development is still possible even in the absence of mature T and B lymphocytes.

## 2. CII-Specific Antibodies

**Figure 1:**
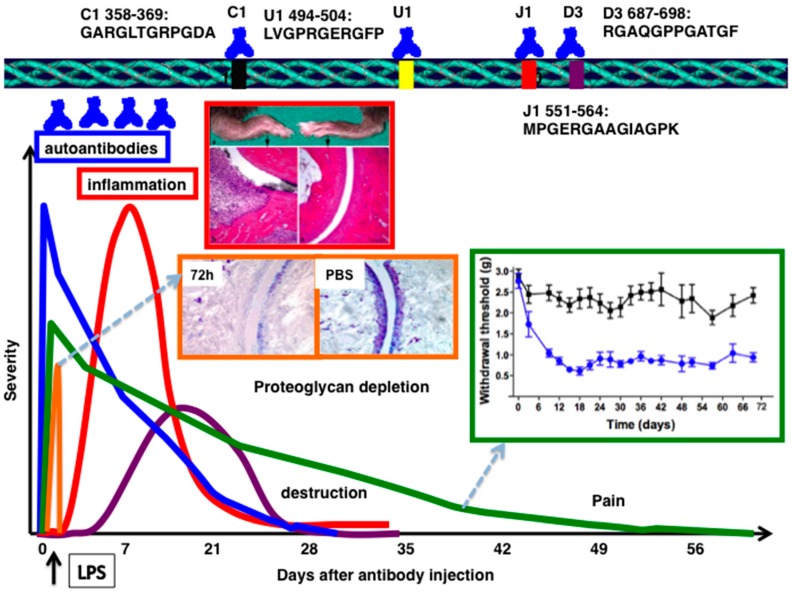
Schematic diagram of acute form of collagen antibody induced arthritis. Autoantibodies binding to well defined epitopes are transferred at day 0, followed by injection of lipopolysaccharide from *E. coli* 05:B55 as the secondary stimulus at day 3. Significant level of proteoglycan depletion was observed 72 h after antibody injection. Inflammation (red and swollenness) and, cartilage and bone erosions between arthritis and control mouse are shown. HE stained joint morphology of arthritis and control mice. Magnification, 10×. Pain (withdrawal threshold levels) started even before inflammation began and prolonged even after resolution of inflammation. Dotted arrows indicate the inserted figures.

Germ line encoded antibodies are important in the pathogenesis of antibody mediated autoimmune diseases [32]. Genetic control of autoantibody responses [33,34] and the association of epitope-specific antibody response with specific *VH* alleles were identified earlier [35]. Antibodies either directly or as constituents of immune complexes, play a central role in triggering inflammation in a number of autoimmune diseases [6,36]. In experimental arthritis, disease can be passively induced in naive mice using serum from arthritic mice [27,37], RA patients [38,39], with a combination of CII specific mAbs [40,41,42,43,44] or single mAb [45]. Arthritis produced by passive transfer of CII mAb, so called collagen antibody induced arthritis (CAIA), resembles actively induced CIA, with a much more rapid onset (24–48 h), but in acute form (Figure 1). LPS (ligand for toll-like receptors, TLR4/TLR2) [41,46] or lipomannan (ligand for TLR2) [47] enhances the incidence and severity of the antibody initiated disease by decreasing the threshold for arthritis induction. Disease susceptibility is independent of MHC alleles [27,42] and severe combined immunodeficient (SCID) mice developed arthritis [48], as well as T or B cell deficient mice [49], but the T and B cell double deficient mice had less severe arthritis [49], suggesting a regulatory role for these cells at the effector level [50,51,52]. CAIA is an acute arthritis that is triggered by antibody binding and neutrophils/macrophages, but bypassing the adaptive immune responses.

For CAIA induction, IL-1β, TNF-α and MIP-1α are required, but not IL-6 [48]. IL-4 [53,54] and IL-10 [55] promoted the disease. Several complement components and their receptors [28,56,57,58,59,60,61] are involved. The complement factor 5 (C5) break down product, C5a is the most potent anaphylatoxin and a powerful chemo-attractant for neutrophils and monocytes, with the ability to promote margination, extravasation, and activation of these cells [62]. C5a levels are markedly elevated in the synovial fluids of patients with RA [63], and a selective C5a receptor antagonist is inhibitory to immune complex–induced inflammation [64]. Hence, C5a plays a crucial role in antibody mediated arthritis [65] and a recombinant vaccine, which induced C5a-specific neutralizing antibodies attenuated CAIA development [66]. Similarly, a fusion protein containing synovial-homing peptide and anti-C5 neutralizing antibody, which specifically targeted inflamed joints attenuated antibody initiated arthritis [67]. Presumably, inflammatory cell recruitment to the joint by C5a or by other complement-induced chemotactic factors are required for the disease initiation.

**Figure 2:**
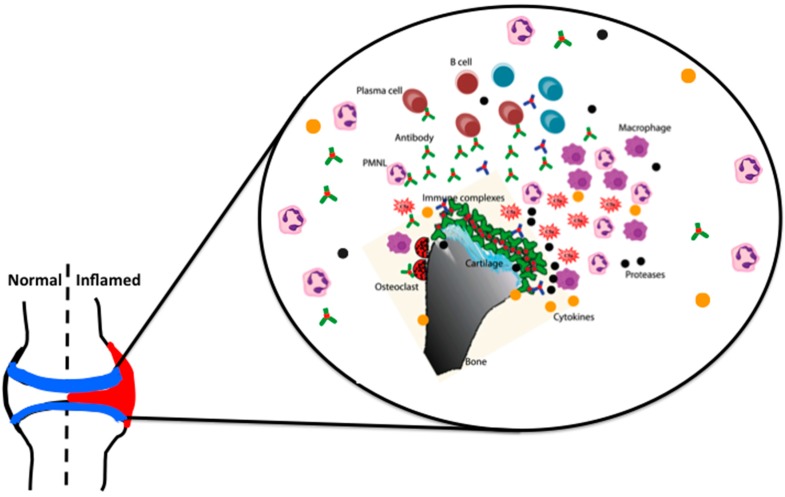
IgG dependent effector phase of arthritis. Binding of antibodies to epitopes present on the cartilage surface forms immune complexes leading to the activation of complement cascades and formation of anaphylatoxin, C5a, which attracts immune cells to the inflammatory foci. Antibodies also interact with FcγR bearing granulocytes, which secrete pro-inflammatory cytokines and proteases damaging cartilage and bone.

Interestingly, C5a binding to C5aR induces the expression of activating FcγRIII, while down modulating inhibitory FcγRII on macrophages, which demonstrates how these two key components of acute inflammation can interact with each other in vivo [68]. Mice lacking the common γ-chain of FcRs are highly resistant [45,69] to CAIA, but are only partially resistant in FcγRIII deficient mice [69]. The absence of FcγRII in DBA/1 mice exacerbates the disease [45], but not so in the BALB/c background [69]. More rapid and severe arthritis was observed with an injection of single anti-CII antibody in FcγIIa transgenic mice [70]. Recent observations also highlight the difference in effector functions of IgG Fc engaged to the complement components and FcγRs [71]. There are several factors that could influence the relative contributions of complement versus FcR dependent inflammatory pathways to the immune complex-triggered inflammatory responses. These include antibody isotype, titer as well as the site of immune complex deposition. With respect to Ig isotype, FcR mechanisms could predominate with immune complexes comprised of non-complement-fixing antibodies or after deposition in sites with abundant resident FcR-bearing inflammatory cells. Conversely, complement-driven inflammation may dominate when immune complexes containing Ig-constant regions poorly bound by FcR or when leukocytes must be attracted to an inflammatory site. In addition, antibody titer may influence the humoral pathways of inflammation [72] and subsequent antibody synthesis by feedback regulation [73]. It has also been shown that C5a can down modulate TLR4 induced immune responses [74], indicating the complexity of interactions occurring during antibody initiated inflammation. In essence, IgG mediated inflammation is mainly dependent on age, sex, FcγRs, complement factors, cytokines (IL-1β, IL-4, IL-10, TNF-α, IFN-β and -γ), chemokines, neutrophils, macrophages, different types of proteases, and other inflammatory mediators, like prostaglandins, leukotrienes, etc. [75,76,77] (Figure 2).

Interestingly, apart from the above described inflammatory phase, antibodies could be pathogenic to the cartilage independent of inflammatory cells and factors [78]. Anti-CII antibodies could be pathogenic to chondrocytes, even in the absence of inflammatory mediators, like involvement in impaired cartilage formation [79], strong inhibition of collagen fibrillogenesis [80], and disorganization of CII fibrils in the extracellular matrix (ECM) with or without increased matrix synthesis [81]. In addition, these pathogenic monoclonal antibodies (mAbs) also induce deleterious effects on cartilage [82,83,84] and inhibit CII self-assembly, which suggests that pathogenic antibodies could possibly interfere with the crucial epitopes at sites essential for the stabilization of the polymeric CII fibrils, leading to disturbances in the integrity of the cartilage matrix. Hence, it is plausible that autoantibodies after binding to the cartilage could initiate unwinding of the triple helical structure of CII, which in turn could lead to proteoglycan depletion [85], allowing more enzymes, inflammatory cells to penetrate into the cartilage architecture to induce further damage. But, direct evidence for these suggested initial pathological events is still not available. Surprisingly, instead of LPS or lipomannan, when mannan from *Saccharomyces cerevisiae* was used as the secondary stimulus after anti-CII antibodies transfer, chronic arthritis phenotype developed in mice having low levels of reactive oxygen species [86] suggesting that under certain in vivo conditions, antibodies could also contribute to chronic disease manifestations and disease relapses in RA.

## 3. COMP-Specific Antibodies

Cartilage oligomeric matrix protein (COMP) is a structural cartilage protein synthesized by chondrocytes and composed of 5 identical subunits, with disulfide bonds near the N-terminal, and with a globular domain at the C-terminal end [87,88]. Immunization with COMP leads to induction of arthritis in rats [89] and mice [90]. Polyclonal antibodies binding to COMP upon passive transfer induced arthritis, albeit at a lower level of severity [90] as compared to CAIA. Subsequently, mAbs to COMP were generated and shown to induce arthritis in mice [91]. In addition, anti-COMP mAbs enhanced arthritis when co-administered with a sub-arthritogenic dose of CII-specific mAb [91].

## 4. Anti-GPI Antibodies

The F1 progeny (KBN) of the KRN TCR (recognizing bovine RNase presented by *A^k^*) transgenic mice and the non-obese diabetic (NOD) mice carrying MHC class II allele *Aβ^g7^* spontaneously developed severe peripheral arthritis beginning at about three weeks of age [92]. T and B cell autoimmunity to the ubiquitous glycolytic enzyme glucose-6-phosphate isomerase (GPI) is the deriving force in this disease model [93]. The KRN TCR recognizes a peptide derived from GPI (residues 282–294), in the context of Aβ^g7^ [94]. After the initiation, the disease proceeds due to the presence of high levels of anti-GPI antibodies that are present in the KBN serum. Injection of recombinant hGPI [95] or hG6PI (325–339) peptide [96] induced arthritis in mice.

Naïve mice injected with KBN serum [97], affinity-purified anti-GPI antibodies [93], or a combination of two or more anti-GPI mAbs [98] induced arthritis. Purified anti-GPI antibodies transferred into the mice localized specifically to distal joints in the front and rear limbs within minutes of injection, saturated within 20 min and remained localized for at least 24 h [99], and the accumulation of immune complexes seems to be possible due to a lack of decay-accelerating factor (DAF) in this tissue [100] and caused macromolecular vasopermeability localized to joints, thus augmenting its severity [101]. The predominant isotype of the antibodies that are present in the KBN serum is γ1 and severe arthritis is maintained if repeated injections of serum are given [97]. Degranulation of mast cells was apparent within an hour [102] and an influx of neutrophils was prominent within 1–2 days [103]; synovial hyperplasia and mononuclear cell infiltration, with pannus formation and erosions of bone and cartilage, began within a week [97,103].

Similar to CAIA, arthritis caused by KBN serum transfer is *MHC* independent. Also, T and B cells are not required since arthritis developed in recombination activating gene 1 (*RAG1*) deficient mice [97] but IL-17-producing T cells can augment this autoantibody-induced arthritis [104]. A single injection of anti-GPI antibody caused prolonged and more severe arthritis in B cell-deficient KBN mice [97]. Mice depleted of neutrophils using anti-Gr-1 antibodies are resistant [103] and neutrophil FcγR, C5aR, and CD11a/LFA-1 are critical components [105]. Interestingly, CpG-oligodeoxynucleotides induced cross talk between CD8α^+^ dendritic cells and NK cells, which resulted in the suppression of neutrophil recruitment to the joint [106]; mice lacking macrophage-like synoviocytes (op/op) are not susceptible [107]. Similarly, mice that were depleted of macrophages by clodronate liposomes were completely resistant. Reconstituting these mice with macrophages from naive animals reversed this resistance [108]. Intravenous immunoglobulins (IVIG) induced expression of FcγRIIB in macrophages but not in neutrophils protected the mice from the disease [107]. Mice having mutations in the stem cell factor (SCF) receptor, c-kit (*W/Wv*) or its ligand, SCF (*Sl/Sld*), leading to mast cells deficiency, are resistant, and susceptibility can be restored by reconstitution with mast cell precursors [102,109]. Subsequently, it was shown that mast cells contribute to the antibody initiated arthritis via IL-1 [110]. TNF-α and IL-1R, but not IL-6 deficient mice were resistant to disease induction [111,112], but TNFR1 and TNFR2 deficient mice were susceptible [113]. IL-4 is dispensable [114] and a genetic polymorphism in IL-1β gene was shown to be of importance [115]. Gene-disrupted or congenic mice were used to delineate the roles of complement components: factor B, C3, C5 and C5aR are essential, but not C1q, C4, MBL-1, C6, CR1, 2, and 3 [113]. Thus, it has been concluded that activation through the alternative pathway leading to the generation of C5a is important in the serum transfer arthritis. Mice lacking the common γ-chain of FcRs are more resistant than those lacking only FcγRIII [113]. But, different results were obtained with FcγRII deficient mice, either they had no effect [113], or they had an earlier onset and greater severity of disease [109]. The neonatal MHC-like FcR (FcRn), associated with the half-life of transferred antibodies, is required [116]. NKT cells promoted this antibody-mediated inflammation [117]. Interestingly, IVIG treatment or ant-murine albumin antibodies protected mice against KBN serum induced arthritis [118], suggesting the importance of antibody-FcR interactions in arthritis pathogenesis.

## 5. Immune-Complex Mediated Arthritis

Immune-complex arthritis (ICA) was elicited in naive mice using a non-self-antigen [119]. Mice injected intravenously with heat-inactivated polyclonal rabbit anti-lysozyme serum, followed by an injection with poly-L-lysine-coupled lysozyme in the joint developed arthritis. Disease featuring a massive influx of neutrophils is evident within a day and wanes over the course of a week. Antigen is deposited on the articular surface, presumably in complex with specific antibody [119]. Local depletion of macrophage-like synoviocytes prevents disease [120]. IL-1 is required for inflammation and cartilage destruction, but TNF-α may be dispensable. In this model, FcγRIII is required for inflammation and cartilage breakdown, but FcγRI seems to be only important in cartilage loss [121], whereas IFN-γ bypasses the dependence on FcγRIII [122]. FcγRII plays a suppressive role, since inflammation and cartilage breakdown are enhanced in FcγRII deficient mice [121]. Chondrocyte death in FcγRI^−/−^ mice was completely abrogated, whereas matrix metalloproteinases (MMPs) mediated cartilage destruction was significantly diminished [121]. Local adenoviral overexpression of IFN-γ in the knee joint prior to the onset of IC-mediated arthritis aggravated severe cartilage destruction. IFN-γ stimulated ICA showed pronounced chondrocyte death that was also completely mediated by FcγRI [123]. Thus, during ICA, synovial macrophages seem to be the dominant factor in the induction of severe cartilage destruction [124].

## 6. Anti-Citrullinated Peptide/Protein Antibodies

Several citrullinated autoantigens (α-enolase, fibrinogen, filaggrin, vimentin, and CII) are used as targets of ACPAs in the diagnostic assays [125]. Around 70% of RA patients sera contain antibodies binding to cyclic citrullinated peptides (CCP2), and these ACPAs are reported to be associated with more severe arthritis [126]. ACPAs are included as one of the classification criteria for RA by American College of Rheumatology/European League Against Rheumatism (ACR/EULAR) consortium [127]. ACPAs are present in the RA sera decades before the onset of clinical disease [128], possibly suggesting that the triggering for autoimmunity may occur at other locations in the body than the joints [129]. Prior to arthritis onset, epitope spreading [130], avidity maturation [131], and changes towards a pro-inflammatory Fc glycosylation phenotype [132] occurs in the ACPA response.

ACPAs activate osteoclasts [133], leading to bone loss even before the onset of clinical disease [134] and the glycosylation status of IgG determines osteoclast differentiation and bone loss [135]. Thus, autoantibodies could have direct influence on osteoclastogenesis by binding to certain activating FcγRs present on immature osteoclasts leading to enhanced osteoclast generation and bone destruction [136]. Binding of ACPAs to osteoclasts releases IL-8, leading to bone erosion [137] and pain [138], which in turn, could lead to pro-inflammatory processes [139]. Furthermore, ACPAs induce macrophages to secrete TNF-α, mediate activation of complement cascades [140], and FcγRIIa-dependent activation of platelets [141]. ACPAs are also shown to be pathogenic in experimental arthritis [142,143]. Hence, it is plausible that ACPAs may play a crucial part in RA pathogenesis [144].

## 7. Antibody Induced Pain

Autoantibodies binding to target tissues can induce pain through Fc, Fab-dependent mechanisms [145] possibly via inflammatory mediators like high mobility group box-1 protein (HMGB1) [146] or chemokines released from osteoclasts [138]. Arthralgia in RA patients’ may precede joint inflammation, may not correlate with the degree of inflammation, and may persist even after successful treatment of inflammation. In this context, KBN serum transfer induced persistent pain and TNF-α/prostaglandin inhibitors attenuated the allodynia induced during inflammation [147]. Experiments with CII-specific pathogenic IgG antibodies demonstrated time-dependent prostaglandin and spinal glial contribution to antibody-induced pain [148]. Spinal HMGB1 also contributes to nociceptive signal transmission via the activation of TLR4 in antibody induced inflammation [146].

## 8. Protective Autoantibodies

Interestingly, not all the antibodies are pathogenic in nature. Some of them could be protective, which suggests the possible regulation at the effector level of arthritis. One of the anti-CII antibodies, named CIIF4 binding to the CII epitope, F4 (ERGLKGHRGFT, amino acids Gly926-Phe936) has a protective role against arthritis, when given in combination with arthritogenic antibodies [85,149]. Cartilage explant studies showed that CIIF4 penetrated the extracellular matrix during culture, remained bound to the tissue [82], induced negligible loss of proteoglycan, minimal chemical changes in the composition of the matrix [85], and allowed matrix regeneration, which required viable chondrocytes [150]. Similarly, one of the ACPA mAbs binding to citrullinated fibrinogen [132,151] was found to be protective [152]. However, the mechanisms (for example, steric hindrance for pathogenic antibody binding to the cartilage, blocking of MMP cleavage sites and/or having protective IgG N-glycome profile) of antibody protection are still not clear.

## 9. Targeting IgG to Treat Antibody Dependent Pathologies

At the effector level of arthritis, apart from targeting effector molecules, like C5 [67,153] and its break down product C5a [65,154,155], receptors (FcRs [156], TLRs [157]), transcription factors [158,159]), and cytokines, using different strategies and drugs [160,161,162], methods for direct targeting of pathogenic IgG antibodies could be attractive and optimal for therapeutic applications.

IgG molecules at Asn-297 of the CH2 domain of IgG Fc part are glycosylated with variable galactosylation and limited sialylation [163]. Changes in N-glycome alter Fc conformation with direct effects on IgG effector functions [164,165] and have important immunoregulatory functions [166]. For example, increasing afucosylated glycoforms by glyco-engineering have significantly increased the cell mediated cytotoxicity of the target bound anti-CD20 antibody [167]. It is clear that sialylation of the of the Fc fragment confers anti-inflammatory properties [168,169]. Anti-inflammatory property of intravenous IgGs (IVIGs) is mainly attributed to sialylated glycans present in the Fc part of IgG [169,170]. Abrogation of the arthritis activity of KBN sera was observed when sialic acids attached to the penultimate galactose of IgG Fc by α2,6 linkages were cleaved using sialidase or after administration of sialic acid enriched Fc fragments [171]. Sialylated Fcs bind to a specific C-type lectin receptors, SIGN-R1 expressed on macrophages [172], leading to the up-regulation of the inhibitory FcγRIIb on inflammatory cells and inhibition of autoantibody initiated inflammation [173,174] via production of IL-33 and, IL-4 [175] acting on IL-4α [176]. Interestingly, sialylation of anti-CII antibodies and ACPAs attenuates arthritogenic activity and leads to suppression of CIA [177]. Recently, several methods have been developed to modulate the glycan pattern of an antibody for therapeutic benefits (for recent review, see [178]).

**Figure 3:**
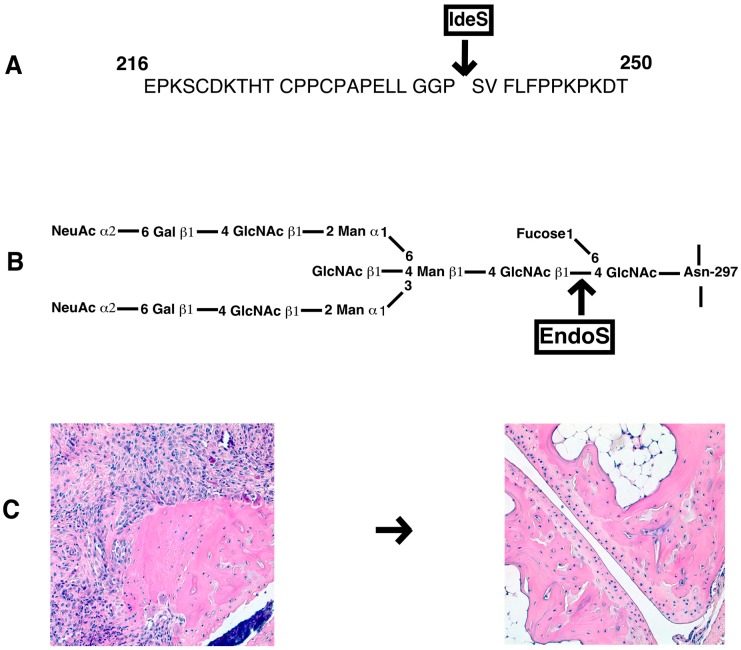
Bacterial enzymes as therapeutics for IgG dependent diseases. *Streptococcus pyogenes* secreted IdeS (**A**) cleaves IgG molecules at the hinge region and EndoS (**B**) cleaves N-linked carbohydrates specifically present on Fc region. Arthritis is ameliorated either after IgG-degrading enzyme (IdeS) or Endo-β-*N*-acetylglucosaminidase (EndoS) cleavage of pathogenic IgG (**C**). HE stained joint morphology of mouse with and without arthritis after treatment. Magnification, 10×.

Bacterial enzymes to specifically cleave IgG at the hinge region or remove specific carbohydrate moieties linked to the N-glycans of the Fc core polysaccharides could also be used for inhibition of antibody induced inflammation (Figure 3). Endo-β-*N*-acetylglucosaminidase (EndoS) is a member of the GlcNAc polymer hydrolyzing glycosyl hydrolases of family 18-glycosyl hydrolase secreted by group A β-hemolytic *Streptococcus pyogenes*. It exclusively hydrolyses the β-1,4-di-*N*-acetylchitobiose core of the N-linked complex type glycan on Asn-297 of the γ-chains of IgG [179]. EndoS treatment of antibodies did not affect binding of IgG to CII and complement activation, but reduced binding to FcγRs and formation of stable immune complexes [180]. EndoS treatment of KBN serum decreased inflammation induced by anti-GPI antibodies [181]. Similarly, pathogenic potential of IgG molecules were attenuated in other inflammatory conditions as well [182]. EndoS is extremely potent in disrupting larger immune complex lattice formation on the cartilage surface possibly through the destabilization of Fc-Fc interactions [183]. Treatment of mice with EndoS has suppressed many antibody mediated experimental autoimmune diseases (thrombocytopenic purpura [184], arthritis [181], glomerulonephritis [185,186], encephalomyelitis [187], hemolytic anemia [188], and epidermolysis bullosa acquisita [189]). Recent studies also showed that treatment with EndoS reduced Fc/FcγR interactions through Fc deglycosylation, which led to reduction in immune complex-mediated neutrophil activation [190].

Another enzyme secreted by *S. pyogenes* is the IgG-degrading enzyme (IdeS), a cysteine endopeptidase, which cleaves the heavy chains of IgG with a unique specificity [191]. By removing the Fc part from the antigen recognizing Fab, immune responses such as complement activation and Fc dependent effector mechanisms are eliminated. IdeS completely blocked antibody-induced arthritis, reduced CIA disease severity, and inhibited antibody initiated arthritis relapses [192]. Similarly, IdeS is effective in ameliorating other IgG dependent pathologies [182]. Recently, IdeS was shown to reduce/eliminate donor specific antibodies and permitted *HLA*-incompatible transplantation in patients [193]. Interestingly, IdeS can also cleave IgG type B cell receptors, leading to abolished receptor mediated signal transduction and memory B cell activation, temporarily [194].

Thus, glyco-engineering of IgG molecules [195], use of bacterial enzymes to specifically cleave IgG or remove certain carbohydrate moieties [78,182], or blocking the downstream effector pathways [65] to ameliorate IgG dependent pathologies offer new avenues for novel drug development. It is of interest to note that several modifications have been reported that could modulate the therapeutic capability of IgG antibodies [196] and designing of antibodies for improving their therapeutic potency has been reviewed recently [197].

## 10. Conclusions

At the IgG mediated effector level of arthritis, different pathways of complement activation, FcγR engagement, activation of residential, and infiltrated immune cells in the synovia, various cytokine and chemokine secretion are essential for the development of clinical disease. Requirement for the APC derived cytokines, TNF-α and IL-1β for arthritis induction and perpetuation is obvious. Whereas, T cell secreted cytokines could be detrimental or protective to the joints, depending on the phase of the clinical disease and in situ conditions. Effector cells of the innate immune system (neutrophils, macrophages, and mast cells) drawn to the inflammatory foci by different chemokines and chemo-attractants are actively engaged to induce inflammation, inflict damage to the cartilage, and perpetuate the ongoing immune responses by secreting cytokines and proteases. Once the stimuli (pathogenic IgG molecules) are eliminated, the inflammation subsides. However, if epitope spreading and release of unexposed antigens or antigenic modifications in the presence of strong immune stimuli (for example, mannan) are continuing within the joint, it could drive the acute disease into chronic inflammation under certain conditions with a complete disruption of joint architecture. Hence, it would be valuable to dissect the fine specificity of the molecules taking part in the pathogenesis, as well as understanding both the upstream and downstream molecular events that are involved in the antibody mediated disease process for effective development of therapeutic strategies. With the recent advances in our knowledge and techniques in various scientific disciplines, the possibility of developing such novel therapies for RA is all the more promising.
